# Age-related murine hippocampal CA1 laminae oxidative stress measured in vivo by QUEnch-assiSTed (QUEST) MRI: impact of isoflurane anesthesia

**DOI:** 10.1007/s11357-020-00162-8

**Published:** 2020-01-25

**Authors:** Bruce A. Berkowitz, Robert H. Podolsky, Karen Lins Childers, Alexander Gow, Brandy L. Schneider, Scott C. Lloyd, Kelly E. Bosse, Alana C. Conti, Robin Roberts, Ali M. Berri, Emma Graffice, Kenan Sinan, Waleed Eliwat, Yimin Shen

**Affiliations:** 1 Department of Ophthalmology, Visual and Anatomical Sciences, Wayne State University School of Medicine, 540 E. Canfield, Detroit, MI 48201 USA; 2 Beaumont Research Institute, Beaumont Health, Royal Oak, MI 48073 USA; 3 Center for Molecular Medicine and Genetics, Wayne State University School of Medicine, Detroit, MI 48201 USA; 4 Department of Pediatrics, Wayne State University School of Medicine, Detroit, MI 48201 USA; 5 Department of Neurology, Wayne State University School of Medicine, Detroit, MI 48201 USA; 6 John D. Dingell VA Medical Center, Detroit, MI 48201 USA; 7 Deptarment of Neurosurgery, School of Medicine, Wayne State University School of Medicine, Detroit, MI 48201 USA; 8 Department of Radiology, School of Medicine, Wayne State University School of Medicine, Detroit, MI 48201 USA

**Keywords:** Imaging, Free radicals, Reactive oxygen species, Brain

## Abstract

Age-related impairments in spatial learning and memory often precede non-familial neurodegenerative disease. Ex vivo studies suggest that physiologic age-related oxidative stress in hippocampus area CA1 may contribute to prodromal spatial disorientation and to morbidity. Yet, conventional blood or cerebrospinal fluid assays appear insufficient for early detection or management of oxidative stress within CA1 sub-regions in vivo. Here, we address this biomarker problem using a non-invasive MRI index of CA1 laminae oxidative stress based on reduction in R1 (= 1/T1) after anti-oxidant administration. An R1 reduction reflects quenching of continuous and excessive production of endogenous paramagnetic free radicals. Careful motion-correction image acquisition, and avoiding repeated exposure to isoflurane, facilitates detection of hippocampus CA1 laminae oxidative stress with QUEnch-assiSTed (QUEST) MRI. Intriguingly, age- and isoflurane-related oxidative stress is localized to the stratum lacunosum of the CA1 region. Our data raise the possibility of using QUEST MRI and FDA-approved anti-oxidants to remediate spatial disorientation and later neurodegeneration with age in animals and humans.

## Introduction

It is commonly suggested that hippocampal-dependent learning and memory are degraded in aged rodents and humans (Daugherty and Raz 2017; Kadish et al. 2009). Clinically meaningful disruptions in spatial learning and memory (e.g., a loss of goal location based on surrounding landmarks) are also associated with repeated anesthesia, such as isoflurane, a common experience for the older patient (Culley et al. 2003; Lin and Zuo 2011; Safavynia and Goldstein 2019). The spatial confusion in these apparently disparate conditions is commonly proposed to stem from oxidative stress within the mid- and posterior hippocampus (HC, human), or dorsal CA1 (CA1, rodents), which are specialized sub-regions of brain essential for encoding spatial information (Ali et al. 2006; Arimon et al. 2015; Chen et al. 2014; Fanelli et al. 2013; Forster et al. 1996; Frisoni et al. 2008; Hall et al. 2012; Han et al. 2015; Kanamaru et al. 2015; McManus et al. 2011; Moser et al. 1993; Mueller et al. 2007; Navarro et al. 2008; Nicolle et al. 2001; Pratico et al. 2001; Raz and Daugherty 2018; Strange et al. 2014; Tucsek et al. 2014). Importantly, prodromal administration of anti-oxidants preserves cognitive performance during healthy aging and in mice exposed to repeated isoflurane anesthesia (Carney et al. 1991; Clausen et al. 2010; Haxaire et al. 2012; Quick et al. 2008; Raghavendra and Kulkarni 2001; Shetty et al. 2014; Stoll et al. 1994; Wu et al. 2015; Zhang et al. 2018).

The benefits of anti-oxidant therapy, however, have not translated from preclinical studies to clinical practice because of the lack of assays for non-invasively evaluating local treatment efficacy (Raz et al. 2015). “Wet” biopsies are spatially non-specific and tissue biopsies are generally unavailable. Imaging brain oxidative stress and the effects of treatment in vivo has so far required the use of exogenous redox probes that are not FDA approved, limiting such studies to animal models (Bačić et al. 2015; Hall et al. 2012; Hou et al. 2018). The goal of this study is to begin to address the long-standing unmet need to measure oxidative stress in vivo with high spatial resolution using an endogenous biomarker to confirm localized efficacy of anti-oxidant treatment in hippocampus CA1 in both experimental models and, ultimately, in patients.

QUEnch-assiSTed (QUEST) MRI is a sensitive tool for non-invasive mapping of oxidative stress without an exogenous contrast agent (Berkowitz 2018; Berkowitz et al. 2019). The QUEST MRI oxidative stress index is a reduction in spin-lattice relaxation rate R1 (1/T1) after acute anti-oxidant administration that maps the location of excessive production of paramagnetic free radicals (Berkowitz 2018). Most QUEST MRI studies to date have examined oxidative stress in photoreceptor neurons and find agreement with “gold standard” free radical measurements ex vivo when tested in several retinopathy models in mice anesthetized with urethane (Berkowitz 2018). However, our preliminary attempts to apply QUEST MRI to brains in adult mice repeatedly anesthetized with isoflurane were confounded by motion artifacts and other technical difficulties (see Discussion) (Berkowitz et al. 2017). It remains unclear if QUEST MRI can non-invasively detect age- or anesthesia-induced oxidative stress within hippocampus CA1 laminae in vivo.

In this study, we mitigate motion artifacts using imaging based on “periodically rotated overlapping parallel lines with enhanced reconstruction (PROPELLER)” (Berkowitz et al. 2017; Pipe 1999). Also, we test the sensitivity of QUEST MRI to measure hippocampal oxidative stress reported in aged mice, and young mice exposed to repeated isoflurane anesthesia (Carney et al. 1991; Clausen et al. 2010; Haxaire et al. 2012; Quick et al. 2008; Raghavendra and Kulkarni 2001; Shetty et al. 2014; Stoll et al. 1994; Zhang et al. 2018). With careful correction of motion and choice of anesthetic, our data demonstrate QUEST MRI is sensitive to oxidative stress in specific laminae within adult mouse CA1 hippocampus in vivo.

## Material and methods

All animals were treated in accordance with the National Institutes of Health Guide for the Care and Use of Laboratory Animals, the Association for Research in Vision and Ophthalmology Statement for the Use of Animals in Ophthalmic and Vision Research, and Institutional Animal and Care Use Committee authorization. Animals were housed and maintained in 12-h:12-h light-dark cycle laboratory lighting, unless otherwise noted, and supplied with standard rodent chow and tap water ad libitum.

### Animal groups

First, we compared mouse brain R1 maps generated from either typical non-PROPELLER (i.e., Cartesian, *n* = 3) or PROPELLER (*n* = 3) acquisition sequences (not shown in Table 1); these mice were anesthetized with isoflurane during MRI examination. Second, for the repeated isoflurane anesthesia studies, 2-month male C57BL/6 (B6J) mice were bred inhouse from Jackson Laboratories (Bar Harbor, ME) breeders (Table 1). Repeated exposure of isoflurane, a complex I inhibitor, can have prolonged effects on brain tissue including production of hippocampus oxidative stress (Bajwa et al. 2018; Li et al. 2018; Ludwig et al. 2004; Wu et al. 2015; Zhang et al. 2018; Zimin et al. 2018). For this condition, mice were exposed to 5% isoflurane to induce anesthesia, monitored by loss of reactivity to the pedal withdrawal reflex, followed by maintenance of anesthesia with 2.5% isoflurane, delivered through a nose cone, for 15 min. This isoflurane level is in line with that typically used to prepare rodents for further surgical procedures and was approved by veterinary staff at Wayne State University (Lowing et al. 2014). Animals were placed on a heating pad maintained at 37 °C during anesthesia procedures and recovery. Following recovery, a subset of mice were subjected to isoflurane exposure again during MRI examination 6 days later for baseline image acquisition and at 24 h later following anti-oxidant exposure as described in the section below. In a companion study, mice were first anesthetized with isoflurane as above, but with urethane anesthesia during MRI examination. Third, in an aging model of hippocampus CA1 oxidative stress, we compared 2-month and 24-month male B6J mice derived from stocks at Jackson Laboratories, but bred and raised at the National Institute of Aging (B6NIA); NIA policy is to rederive mice from pedigreed stock every 6–7 years (https://www.nia.nih.gov/research/dab/animal-replacement-policy/colony-monitoring-and-history). These mice were anesthetized with urethane during MRI examination.

**Table 1:** Experimental conditions

Groups	Anesthetic during QUEST MRI	Same mouse (*n*)		Different mice (*n*)	
		Baseline	MB/ALA	Saline × 2	MB/ALA
From Jackson Labs					
2 months C57BL/6 (B6J)	Urethane			5	5
2 months B6J, isoflurane (15 min, 1 week prior)	Urethane			9	10
2 months B6J isoflurane (15 min, 1 week prior	Isoflurane	5			
From NIA					
2 months B6NIA	Urethane			7	7
2 months B6NIA	Urethane			6	6

### MRI

The general mouse preparation for 2D MRI is well established in our laboratory (Berkowitz 2018). QUEST MRI following urethane anesthesia accurately assesses retinal oxidative stress in several mouse models (Berkowitz 2018). However, urethane is a terminal anesthetic and only cross-sectional studies are possible. This subgroup had two separate arms. One arm received 1 ml of saline intraperitoneally (IP) at two time points (saline × 2): ~ 24 h and ~ 1 h before acquisition of a T1 data set. The other arm received 1 mg/kg methylene blue (MB, IP, dissolved in saline). MB is an alternate electron transporter that effectively suppresses superoxide generation from mitochondria and various oxidases (Wen et al. 2011). The following day, approximately 1 h before the T1 MRI data collection, MB-treated mice received 50 mg/kg α-lipoic acid (ALA, IP, dissolved in saline and adjusted to pH ∼ 7.4); images were collected from a single slice (− 2 Bregma). ALA is a potent free radical neutralizer (Berkowitz et al. 2015; Berkowitz et al. 2016b; Gomes and Negrato 2014). Unless otherwise indicated, mice were anesthetized with urethane (36% solution intraperitoneally; 0.083 ml/20 g animal weight, prepared fresh daily; Sigma-Aldrich, St. Louis, MO) prior to MRI.

A different set of mice received isoflurane prior to MRI and isoflurane during MRI acquisition to monitor signal changes pre- (baseline) and post-anti-oxidant in the same mouse. Mice were anesthetized with isoflurane (3% induction, 1.2% maintenance) and a baseline T1 data set (as above). Mice were then given either saline or MB (vide supra) 30 min after recovery. The following day, each mouse given either saline or ALA (vide supra) was re-anesthetized with isoflurane, and another T1 data set obtained of the same brain slice as the previous day. In all cases, mice were humanely sacrificed at the end of the study. All experimental conditions and numbers of mice are summarized in Table 1.

In all studies, identical MRI sequences were used, and mice body temperature was regulated by a water bath that is integrated into the MRI cradle. T1 2D (collected using a PROPELLER sequence called BLADE (proprietary code) on the scanner used in this study with motion reconstruction) and T2 (multi-slice, collected with Cartesian acquisition) data sets were acquired on a 7 T scanner (Bruker ClinScan) using a receive only 4-element phased array coil. Sixteen T2-weighted images were acquired using a turbo spin echo sequence (repetition times (TRs) 2.05 s; echo time (TE) 12 ms; echo train length 7; number of averages 4; spacing between slices 0.4 mm; slice thickness 400 μm;, 12 × 12 mm^2^, matrix size 192 × 192, in-plane resolution 62.5 μm). To measure T1, images with different repetition times (TRs) were acquired in the following order (number of averages in parentheses): TR 0.15 s (6), 3.50 s (1), 1.00 s (2), 1.90 s (1), 0.35 s (4), 2.70 s (1), 0.25 s (5), and 0.50 s (3). To compensate for reduced signal-to-noise ratios at shorter TRs, progressively more images were collected as the TR decreased. A spin-echo image was collected in a single transverse slice (echo time (TE) = 55 ms, 12 × 12 mm^2^, matrix size 192 × 192, in-plane resolution 62.5 μm, slice thickness 400 μm, turbo factor 9) at each TR; please note that susceptibility changes caused by inhomogeneity’s of the static magnetic field are expected to be nearly eliminated when using a spin-echo acquisition and thus unlikely to contribute to the observed signal. This saturation recovery approach provides precise 1/T1 values over a large range of signal-to-noise conditions and is routinely performed in our laboratory (Berkowitz 2018; Berkowitz et al. 2016a; Décorps et al. 1985; Freeman and Hill 1971; Haacke et al. 1999; Hsu et al. 2009).

### MRI data analysis

Because different hippocampus layers show different contrasts on T1- and T2-weighted images (see, for example, “ImageBoost” in https://scalablebrainatlas.incf.org/mouse/WHS12**)**, comparisons between these two data sets were useful for anatomical identification. Within each T1 data set of 23 slices, images acquired with the same TR were first registered (rigid body) and averaged to generate a stack of 8 images. The averaged images were registered across TRs. Thereafter, 1/T1 maps were calculated by fitting the data to a three-parameter T1 equation:

1 $$ \mathrm{y}=a+b\ast \left(\exp \left(-c\ast \mathrm{TR}\right)\right) $$

where *a*, *b*, and *c* are fitted parameters on a pixel-by-pixel basis using R (v.2.9.0) and in-house scripts. We previously reported that day-to-day variations in R1 can be mitigated by removing slice bias in the 2D data and low signal-to-noise ratio, because the T1 estimate is highly dependent on the signal intensity of the TR 150-ms image and, thus, is imprecise (Chapter 18 in Haacke et al. 1999). By normalizing to the shorter TR, some of the bias can be removed and a more accurate T1 estimate obtained between days. We normalized within and between groups for signal intensity differences by first applying 3 × 3 Gaussian smoothing (performed three times) only to the TR 150-ms image to suppress noise and emphasize signal. The smoothed image was then divided into the rest of the images of the T1 data set (Berkowitz 2018). An in-house R script was used to convert these 7 images into an R1 map.

**Fig. 1:**
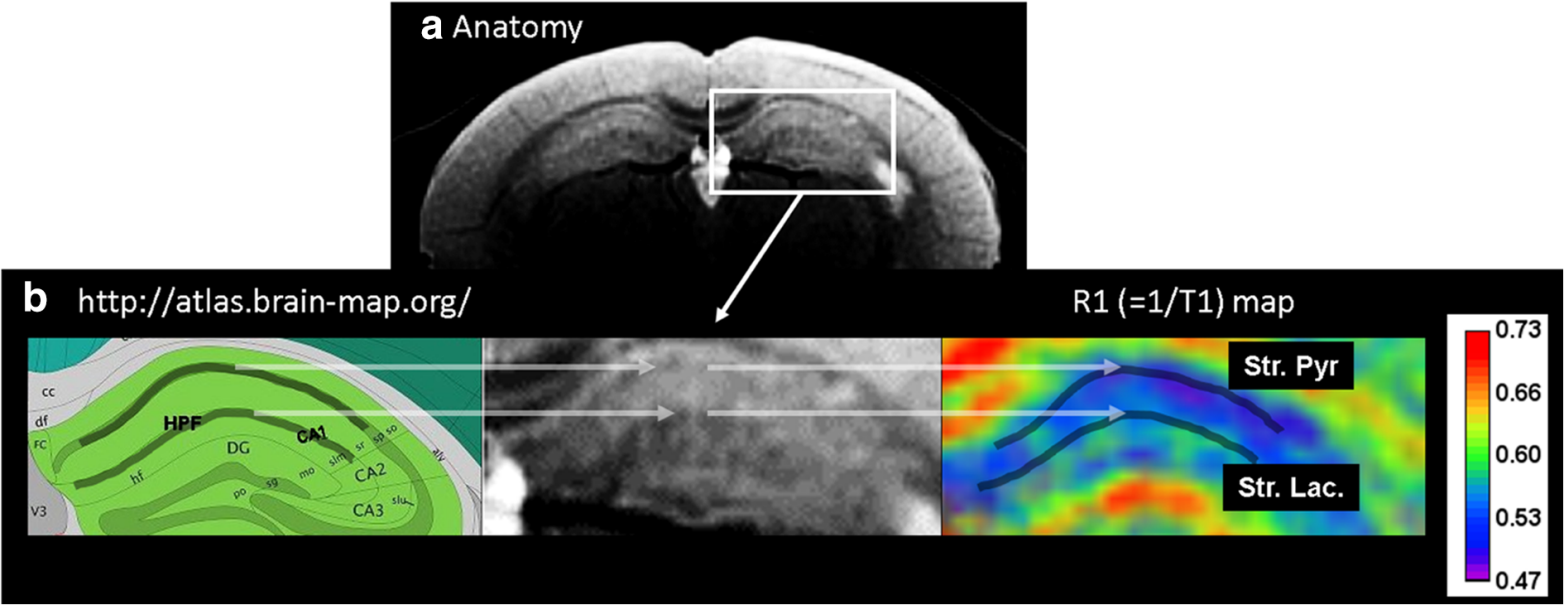
**a** An MRI image illustrates the position of the slice in this study and ROI over the right hippocampus formation (HPF, white box). **b** Identification with the hippocampus CA1 stratum pyramidale (Str. Pyr.) and stratum lacunosum (Str. Lac.) as gray lines drawn on the cartoon at the far left. Calibration bar indicates the range of R1s shown

To analyze the data, first the T1 and same slice T2 images were re-sized to 1440 × 1440 pixels using an ImageJ bilinear interpolation routine to iteratively draw a 5-pixel width segmented line region-of-interest (ROI) on the stratum lacunosum and stratum pyramidale layers as shown in Fig. 1 (Schneider et al. 2012). The R1 map was also re-sized to 1440 × 1440 pixels, but without any interpolation and ROIs applied to extract R1 values from each laminae (Fig. 1). This procedure was performed in triplicate for each ROI per mouse. These three values were used in the linear-mixed model analysis described below.

### Statistical analysis

Data are presented as mean ± SEM. We conducted two separate analyses comparing the different groups of mice in Table 1: (1) a comparison of urethane-anesthetized mice exposed to isoflurane a week before QUEST MRI, and mice exposed to isoflurane a week before QUEST MRI in isoflurane-anesthetized mice; and (2) a comparison of 2-month B6NIA, 24-month B6NIA, and 2-month B6J urethane-anesthetized mice. All data contain repeated measures for each mouse, with at least triplicated and separate measurements for left and right sides of the brain. Further, mice repeatedly exposed to isoflurane were measured before and after being given anti-oxidants. With multiple measurements per mouse, we used linear-mixed models (PROC MIXED and PROC GLIMMIX in SAS) to analyze the data, with each specific model defined by the specific comparisons above. For all analyses, we first evaluated whether any random coefficients other than the random intercept had a large impact on model fit based on the Akaike and Schwarz Bayesian information criteria (AIC and BIC). We also evaluated heterogeneity in the residual variance and in the random effects among groups using AIC and BIC. More complex models were only favored when both AIC and BIC showed a reduction of at least 10. All fixed effects were evaluated using the likelihood ratio test. A significance level of 0.05 was used for tests of main effects, while interactions were tested using a significance level of 0.10 due to these tests having less power. All non-significant interactions were removed except for the group by anti-oxidant interaction to obtain the final model.

The final model for the comparison of urethane- and isoflurane-anesthetized mice included random coefficients for intercept, side, anti-oxidant exposure, and the interaction of side and anti-oxidant for each mouse nested within anesthetic. This model also included the fixed effects of anesthetic, side, anti-oxidant exposure, and all interactions among these main effects prior to testing fixed effects.

To compare the age groups, we initially analyzed all data together, evaluating the random coefficient for side and heterogeneity in the residual variance among groups using the AIC and BIC. These fit statistics indicated a large improvement of fit by including heterogeneity in the residual variance as well as by including the random coefficient for side. However, the resulting model had a large gradient and missing standard errors for some variances. As such, we decided to analyze the two sides separately. The final model for the comparison of age groups included a random intercept for each mouse nested within age group and a separate residual variance for each group. This model also included the fixed effects of anti-oxidant exposure, age group, and the interaction between anti-oxidant and age.

## Results

### Motion artifacts

**Fig. 2:**
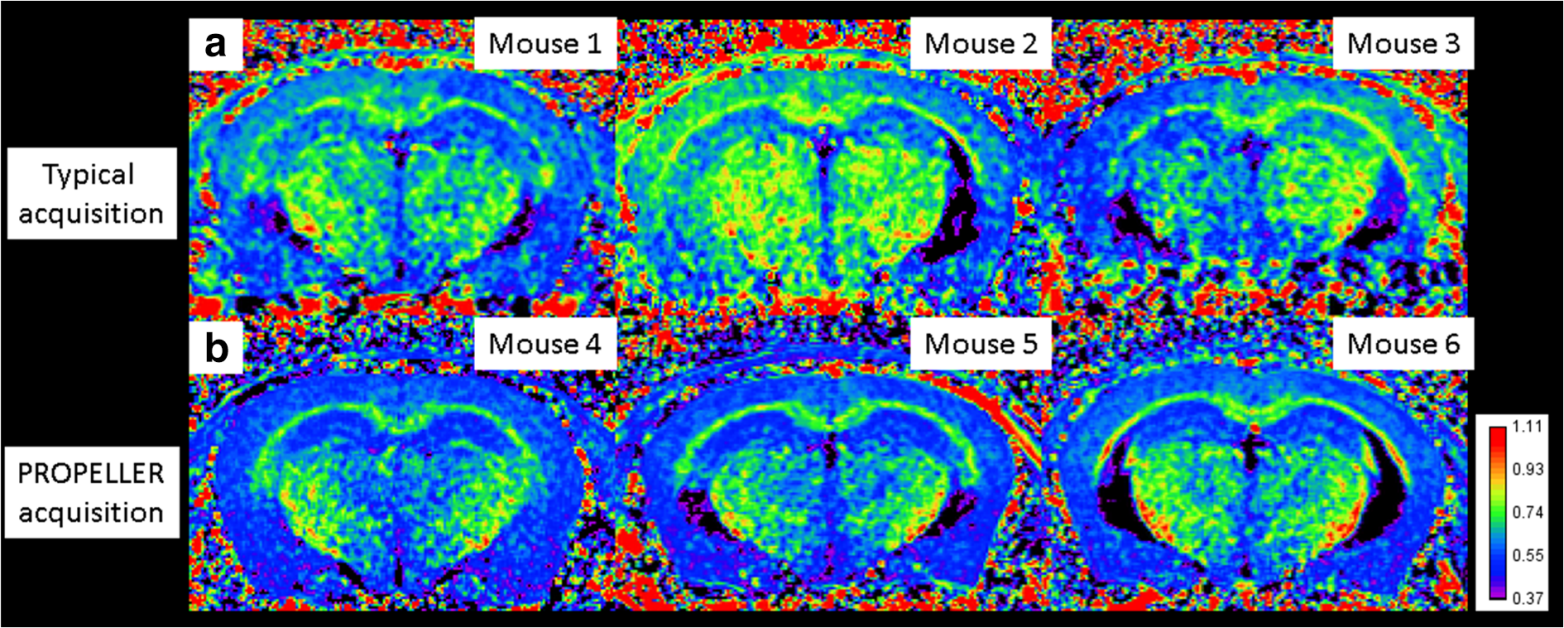
Representative images showing R1 maps from data collected with **a** a typical acquisition sequence or **b** a periodically rotated overlapping parallel lines with enhanced reconstruction (PROPELLER) sequence (called BLADE on the system used in this study). In all cases, post-processing normalization to the TR 150-ms image was performed to suppress B1 inhomogeneity artifacts, and coil and slice bias (Berkowitz et al. 2019). Calibration bar indicates the range of R1s shown

**Fig. 3:**
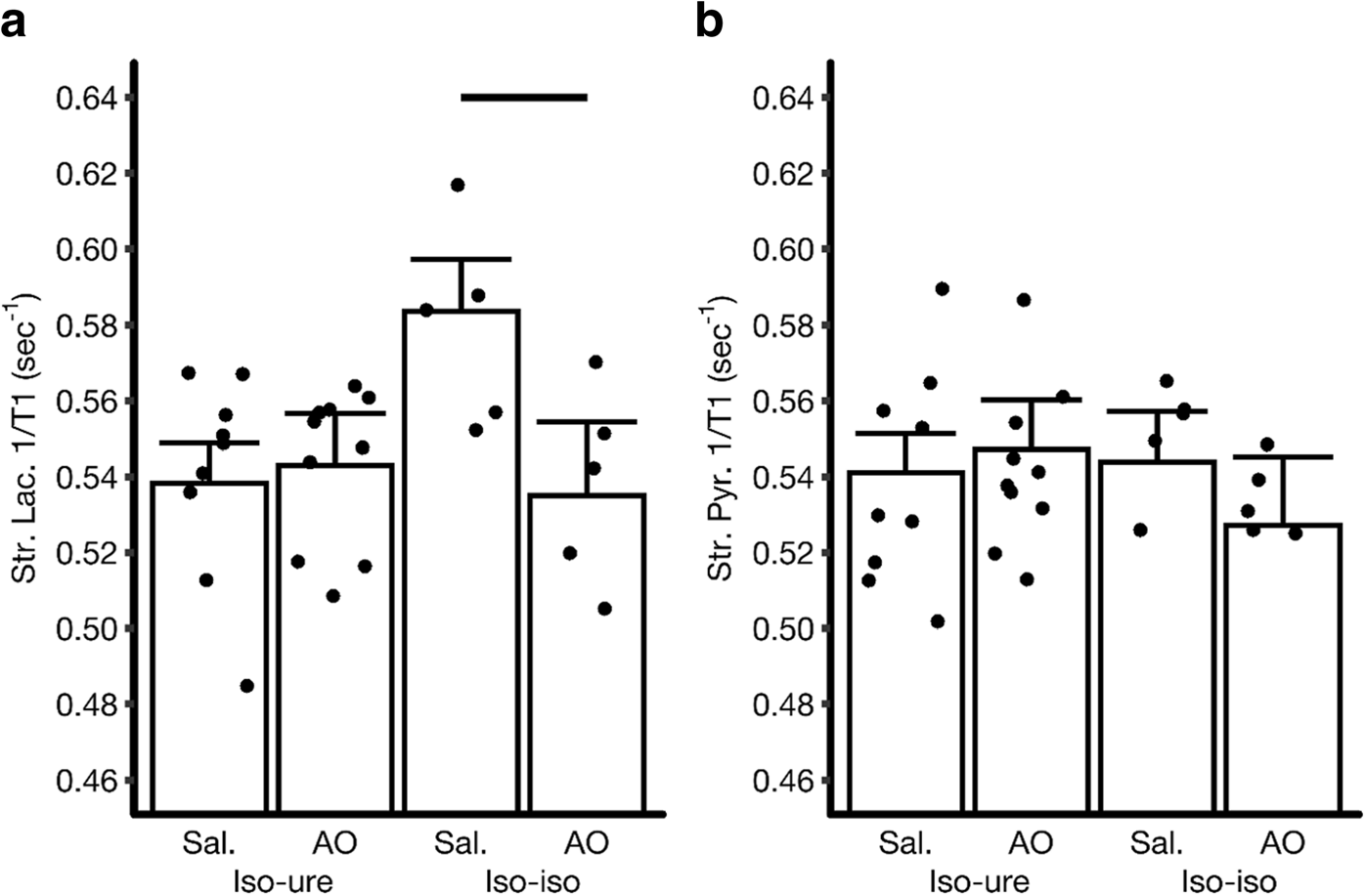
Modeled R1 mean **a** stratum lacunosum (Str. Lac.) or **b** stratum pyramidale (Str. Pyr.) (illustrated in Fig. 2) for different B6J groups exposed to isoflurane a week before QUEST MRI in urethane-anesthetized mice (iso-ure, left two bars) vs. mice exposed to isoflurane a week before QUEST MRI in isoflurane-anesthetized mice (iso-iso, right two bars). The number of animals in each group is presented in Table 1. Averages of R1 for each mouse are shown by circles. Error bars: SEM. Please note that the SEMs shown are based on a statistical modeling of the data. As such, the SEM is similar across groups. Reductions in R1 with anti-oxidants are considered to be an index of oxidative stress and significant changes are indicted with a horizontal black bar (*P* < 0.05)

**Fig. 4:**
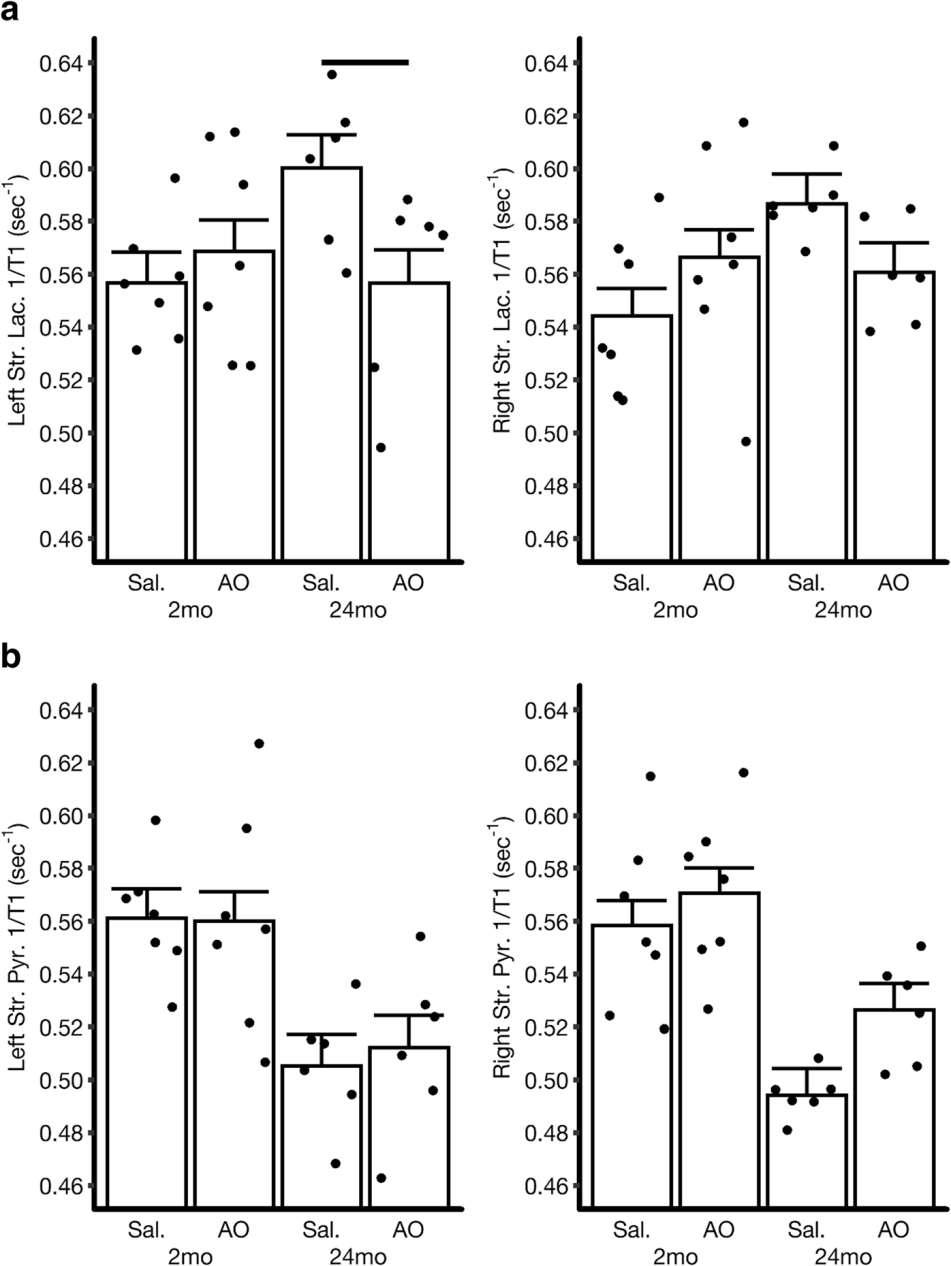
Modeled R1 mean **a** left and right stratum lacunosum (Str. Lac.) or **b** stratum pyramidale (Str. Pyr.) (illustrated in Fig. 2) for different B6NIA groups aged either 2 months (left two bars) or 24 months (right two bars) from QUEST MRI scans of urethane-anesthetized mice. The number of animals in each group is presented in Table 1. Averages of R1 for each mouse are shown by circles. Error bars: SEM. Please note that the SEMs shown are based on a statistical modeling of the data. As such, the SEM is similar across groups. Reductions in R1 with anti-oxidants are considered to be an index of oxidative stress and significant changes are indicted with a horizontal black bar (*P* < 0.05)

R1 maps generated with a PROPELLER sequence are sufficient to correct motion artifacts compared with a standard acquisition sequence, as is evident in the representative images in Fig. 2. Anatomical boundaries are easier to identify in PROPELLER images, which are visibly free of gross and artifactual asymmetries seen in R1 maps compared with those acquired using the typical approach (Fig. 2). The precision obtained using PROPELLER is supported by the results in Figs. 2, 3, and 4 below.

### Repeated isoflurane-evoked hippocampus CA1 oxidative stress in vivo

As shown in Fig. 3, pre-exposure to isoflurane 1 week prior to QUEST MRI using urethane anesthetic does not show oxidative stress in either strata lacunosum (Fig. 3a) or pyramidale (Fig. 3b). In contrast, evidence for hippocampus CA1 oxidative stress is suggested in animals pre-exposed to isoflurane 1 week prior to QUEST MRI examination performed using isoflurane (i.e., R1 is higher at baseline than in animals given urethane (*P* = 0.0205; not shown on graph for clarity)) and confirmed by a reduction in R1 in a second MRI study performed using isoflurane after giving anti-oxidants. In particular, CA1 oxidative stress is apparent in CA1 stratum lacunosum (Fig. 3). This result is statistically invariant between left and right hemispheres and the data are averaged for each mouse. We note that showing both right and left comparisons would only be appropriate if we were showing the raw means and not those estimated from the final statistical model. This is because the modeled differences among the groups would be identical for both sides; adding these results would create a more complicated figure that does not contain new information.

### Age-related CA1 oxidative stress in vivo

Twenty-four-month B6NIA mice showed a suggestion of oxidative stress in CA1 stratum lacunosum since R1 is higher at baseline than in 24-mo B6NIA mice (left hemisphere, *P* = 0.0082; right hemisphere, *P* = 0.0111; not shown on graph for clarity). Oxidative stress was then established by a reduction in R1 after anti-oxidant as measured by QUEST MRI under urethane anesthesia (Fig. 4; see Discussion). Statistical significance is reached in the left stratum lacunosum but not the right, with the estimated effect of anti-oxidants on the right (− 0.026, 95% CI: − 0.059, 0.007) being about ½ of that on the left (− 0.044, 95% CI − 0.080, − 0.007). In contrast, stratum pyramidale does not show oxidative stress in either hemisphere (i.e., no reduction in R1 with anti-oxidants). Also, no effect of vendor (*P* > 0.28) is found when comparing R1 values ± anti-oxidants in 2 months B6J versus B6NIA mice (not shown).

## Discussion

The goal of this study is to develop a viable QUEST MRI approach to measure layer-specific murine hippocampal CA1 oxidative stress in vivo. The present work focuses on addressing problems in our earlier report of QUEST MRI for measuring hippocampal formation oxidative stress (Berkowitz et al. 2017). The first problem is that substantial motion artifacts in the images of the hippocampal formation collected using a standard acquisition yield relatively few animals with usable data (Berkowitz et al. 2017). Here, we show that a PROPELLER acquisition sequence solved this problem in the adult mouse brain (Fig. 2) (Berkowitz et al. 2019). The second problem is a paradoxical finding of hippocampus CA1 oxidative stress in control mice repeatedly exposed to isoflurane. This problem was likely masked in our initial report by combining control groups and/or by the motion artifacts that are clearly evident in Fig. 2. In addition, isoflurane-evoked oxidative stress in the hippocampus of control mice can confound interpretation of QUEST MRI data in experimental models (Ni et al. 2017; Zhang et al. 2018; Zimin et al. 2018). Here, we find limiting isoflurane to a single pre-examination exposure followed by urethane anesthesia is useful for measuring the presence (in experimental groups) or absence (in controls) of oxidative stress in the murine brain in vivo (Fig. 3). These data support and extend the suitability of urethane for QUEST MRI first reported in experimental retina studies (Berkowitz 2018), a likely result of urethane’s ability to minimally alter, for example, functional connectivity, and autonomic and cardiovascular systems (Hara and Harris 2002; Paasonen et al. 2018). A related problem was our prior use of a corpus callosum ROI near hippocampus CA1 (Berkowitz et al. 2017). A recent study identifies profound and prolonged changes in corpus callosum microstructure after isoflurane anesthesia in control mice—a clear confound in the interpretation of R1 (Bajwa et al. 2018; Berkowitz et al. 2017; Hirata et al. 2011; Li et al. 2018; Xu et al. 2018; Zhang et al. 2018; Zimin et al. 2018). Correcting motion artifacts and avoiding repeated isoflurane anesthesia increases the reliability of QUEST MRI as a method for measuring oxidative stress in murine hippocampus CA1 laminae in vivo*.*

To strengthen our statistical power, our a priori design was to examine only two hippocampus laminae for oxidative stress. These two layers were chosen based on studies showing that stratum lacunosum has relatively greater energy metabolism (e.g., glucose utilization, and succinate dehydrogenase and cytochrome c oxidase activity), and more prominent vascularity than stratum pyramidale (Borowsky and Collins 1989; Gulyas et al. 2006; Kann 2016; Kubota et al. 1993; Kugler et al. 1988; Mattiasson et al. 2003; Nicolle et al. 2001; Shimada et al. 1992; Shimada et al. 1994; Shimada et al. 1989; Wang et al. 2005; Wilde et al. 1997).

In contrast to the current study, several ex vivo studies have suggested that the stratum pyramidale is particularly susceptible to developing oxidative stress (Fekete et al. 2008; La et al. 2019; Santini et al. 2015; Stebbings et al. 2016). This apparent disparity with our findings may arise from substantial methodological differences, where the majority of the previous studies utilize dihydroethidium (DHE) staining as a marker for oxidative stress (Du et al. 2013; Michalski et al. 2014; Santini et al. 2015). Ex vivo staining with DHE reveals the cumulative effects of oxidative stress—that of oxidized DNA in the nucleus—even if superoxide is not produced in this organelle (Du et al. 2013; Michalski et al. 2014). Concerns about using DHE, and its derivatives like MitoSox, continue to be raised (Cheng et al. 2018; Xiao and Meierhofer 2019). Also, comparing DHE and QUEST MRI indices may not be justified because DHE measures oxidative damage that builds up over time, in contrast to QUEST MRI which is a snapshot of excessive free radical production. In support of this notion, QUEST MRI results show spatial agreement with data from ex vivo probes such as lucigenin or dihydrodichlorofluorescein (DCF), assays that provide snapshot measurements of free radical production in retinopathy models (Berkowitz 2018). However, we cannot rule out the possibility that oxidative stress was not detected in stratum pyramidale because of its small thickness leading to increased partial volume averaging.

Because of the potentially confounding problems of variable post-mortem intervals for ex vivo assays of oxidative stress and/or damage (above), we have argued that QUEST MRI can be used as a stand-alone in vivo assay of oxidative stress (Berkowitz 2018; Cheng et al. 2018; Xiao and Meierhofer 2019). QUEST MRI results, as noted above, are in agreement with ex vivo lucigenin and DCF assays in retinopathy. Also, the detection of excessive free radical production by QUEST MRI has been confirmed in a phantom study of the xanthine-xanthine oxidase reaction (Berkowitz 2018). Further, the biophysics underlying QUEST MRI is consistent with the expected impact of a continuous and asynchronous production of paramagnetic free radicals on R1 (Berkowitz 2018). Furthermore, the results of the present study demonstrate agreement between QUEST MRI and the extant literature which establish that repeated isoflurane causes excessive production of free radicals in the hippocampus (Hirata et al. 2011; Li et al. 2018; Wu et al. 2015; Xu et al. 2018; Zhang et al. 2018; Zimin et al. 2018). Similarly, the present QUEST MRI data from the 24-month mice are consistent with reports of hippocampal oxidative stress during aging, which is commonly proposed to arise from reductions in anti-oxidant defense mechanisms over time (Ahn et al. 2016; Ali et al. 2006; Antier et al. 2004; Dugan et al. 2009; Fukui et al. 2002; Hall et al. 2012; Haxaire et al. 2012; Lacoste et al. 2017; Nicolle et al. 2001; Stebbings et al. 2016).

In our analyses of oxidative stress in the CA1 of aged mice, the most robust QUEST MRI evidence was found in the left CA1 stratum lacunosum of 24-month-old mice. We were not able to evaluate whether the anti-oxidant response differs between the two sides due to computational problems in fitting an appropriate statistical models when the two sides were combined. The actual anti-oxidant response in 24 month-old mice for the two sides may be similar, and intermediate to estimates obtained separately for the two sides. As such, our study does not provide evidence for age-related lateralization of hippocampus CA1 oxidative stress. Further, QUEST MRI measurements obtained before or after the 24-month time point may show a different spatial patterns with regard to hippocampus CA1 oxidative stress.

The results of this study, and those from the literature, highlight the need for caution when using isoflurane which, when used repeatedly, is not benign as is often assumed (Bajwa et al. 2018; Crystal et al. 2012; Huang et al. 2018; Li et al. 2018; Lin and Zuo 2011; Ni et al. 2017; Paasonen et al. 2018; Wu et al. 2015; Zhang et al. 2018). Isoflurane is known to induce cerebral hyperemia and suppresses neuronal activity (Kehl et al. 2002; Pan et al. 2015; Schroeter et al. 2014; Toyama et al. 2004). More work is needed to investigate variables such as dose, and post-isoflurane duration, to explore how the first isoflurane exposure apparently primes the brain to produce hippocampus CA1 stratum lacunosum oxidative stress in vivo after second and third isoflurane exposures (Fig. 3).

In summary, we developed a QUEST MRI protocol that uniquely measures murine hippocampus CA1 laminae oxidative stress in vivo. This study addresses long-standing shortcomings of conventional assays in experimental studies. We speculate that applications of QUEST MRI in humans will be possible in the future. Since urethane is not clinically translatable, we anticipate that more work will be needed to identify acceptable anesthesia protocols in patients that require sedation for their MRI examination. QUEST MRI is based on an endogenous contrast mechanism and is performed with FDA-approved anti-oxidants and thus is a promising method to facilitate testing of anti-oxidant treatments for mitigating behavioral spatial disorientation, and perhaps other aspects of oxidative stress-associated cognitive dysfunction (Raz and Daugherty 2018).
